# Fabry–Pérot Cavities with Suspended Palladium Membranes on Optical Fibers for Highly Sensitive Hydrogen Sensing

**DOI:** 10.3390/molecules28196984

**Published:** 2023-10-09

**Authors:** Feng Xu, Jun Ma, Can Li, Churong Ma, Jie Li, Bai-Ou Guan, Kai Chen

**Affiliations:** Guangdong Provincial Key Laboratory of Optical Fiber Sensing and Communications, Institute of Photonics Technology, Jinan University, Guangzhou 511443, China

**Keywords:** Fabry–Pérot cavity, hydrogen sensor, optical fiber, palladium

## Abstract

Hydrogen (H_2_) sensors are critical to various applications such as the situation where H_2_ is used as the clean energy for industry or the indicator for human disease diagnosis. Palladium (Pd) is widely used as the hydrogen sensing material in different types of sensors. Optical fiber H_2_ sensors are particularly promising due to their compactness and spark-free operation. Here, we report a Fabry–Pérot (FP)-cavity-based H_2_ sensor that is formed with a freestanding Pd membrane and integrated on a conventional single-mode optical fiber end. The freestanding Pd membrane acts both as the active hydrogen sensing material and as one of the reflective mirrors of the cavity. When the Pd film absorbs H_2_ to form PdH_x_, it will be stretched, resulting in a change of the cavity length and thus a shift of the interference spectrum. The H_2_ concentration can be derived from the amplitude of the wavelength shift. Experimental results showed that H_2_ sensors based on suspended Pd membranes can achieve a detection sensitivity of about 3.6 pm/ppm and a detection limit of about 3.3 ppm. This highly sensitive detection scheme is expected to find applications for sensing low-concentration H_2_.

## 1. Introduction

As a renewable energy source, hydrogen has attracted increasing attention for its potential to replace fossil fuels. However, due to its high diffusion coefficient, low ignition energy, high combustion heat, and wide flammable range (4–75%), there are severe safety risks during the transportation, storage, and use of H_2_ [1,2,3,4,5]. Additionally, H_2_ is colorless and odorless with the smallest molecular weight, which makes it easy to leak but hard to be detected in practical applications [6,7,8]. Therefore, reliable and accurate H_2_ monitoring at low concentrations is extremely important. It is noted that H_2_ can also be used for medical diagnosis in a simple and noninvasive way with breath tests, where the amount of H_2_ acts as indicators of certain digestive problems. Therefore, highly sensitive H_2_ sensors are desired for safe and fast H_2_ detection or monitoring. Over the past decades, various types of H_2_ sensors have been proposed and developed, including electrochemical sensors, micromechanical sensors, resistance sensors, and optical sensors [9,10,11,12]. Among these sensors, optical sensors, especially optical fiber sensors, have shown attractive and promising prospects due to their combined properties of compactness, high sensitivity, good reliability, and anti-electromagnetic interference [13,14]. Optical fiber H_2_ sensors use optical signals as the sensing transducers, which eliminates any potential electrical sparks in the sensing site [15,16,17,18].

One type of optical fiber H_2_ sensor involves integrating a miniaturized FP cavity on top of the end of a single-mode optical fiber. The FP cavity is formed by a short silica capillary with the fiber end as one reflective element and a diaphragm on the capillary as the other reflective element. Such sensors have been widely studied because of their small size, simple structure, high sensitivity, and low cost [19,20,21]. To make the cavity responsive to H_2_, the diaphragm usually consists of Pd, which is a hydrogen-sensitive material with high affinity for H_2_ and good reversibility [22,23,24]. In most cases, the diaphragms usually appear in the form of composite layers with other elastic materials as supporting layers [25,26]. For example, Ma et al. [26] used a hybrid Pd/graphene film to construct the Fabry–Pérot cavity. While the multilayer graphene provides good support for the Pd layer, the stiffness of graphene could result in lower H_2_ sensitivity of the sensor, ≈0.25 pm/ppm. Zhang et al. constructed a similar FP cavity on the fiber tip with UV-curable epoxy [27]. They first transferred a gold (Au) film on top of a capillary with a simple press-and-detach method where UV-curable epoxy was used to bond the gold film to the capillary. A thin Pd layer was then deposited forming a composite Au/Pd H_2_-sensitive film. The sensor shows a good response to H_2_ in the range of 1~3.5%. In another work, Xiong et al. fabricated a FP cavity on top of a fiber tip with a micro-cantilever that is developed by a two-photo polymerization (TPP) method with femtosecond laser printing [28]. Subsequent Pd sputtering enables the cantilever and the FP cavity sensitive to environmental H_2_ changes. In addition, the preparation process of the composite films can be complex, adding to the overall cost of the sensor. Therefore, it is highly desirable to investigate the H_2_ sensing performance of a freestanding Pd thin film without any support materials.

In this study, we report an optical fiber H_2_ sensor that uses a suspended Pd thin film (30 nm in thickness) to form a FP cavity. The Pd film is readily transferred onto a silica capillary that is fused onto an optical fiber. After hydrogen absorption, the expansion of the Pd lattice causes stretching and deformation of the Pd film resulting in shortening of the cavity and blue-shift of the interference spectrum. Then, the H_2_ concentration can be derived from the magnitude of the wavelength shift. The results show that unsupported Pd thin films enable highly sensitive detection of H_2_ at low concentrations.

## 2. Results and Discussions

**Figure 1:**
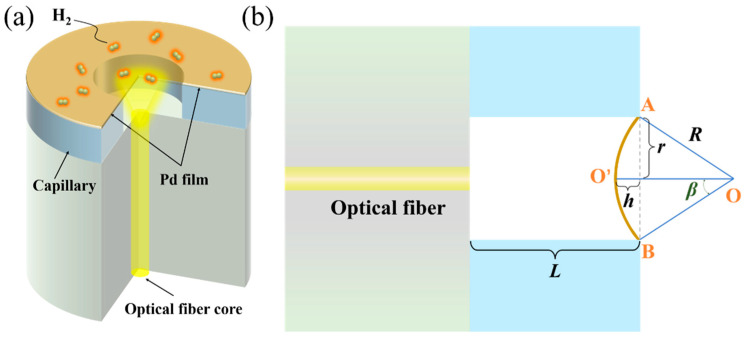
(**a**) Illustration of the proposed hydrogen sensor based on a FP cavity by a freestanding Pd film on an optical fiber. (**b**) The geometrical diagram used to characterize the bending of the Pd film. L is the length of the capillary tube and the initial length of the FP cavity. The remaining symbols in the figure are explained later in the text.

The proposed optical fiber H_2_ sensor is schematically shown in Figure 1a. The main component of the sensor is made from a section of quartz capillary tube that is fusion spliced to a single mode optical fiber. The flat fiber end and the suspended Pd film over the capillary act as two mirrors to form a low finesse FP cavity. When the sensor is exposed to hydrogen, the Pd film absorbs the hydrogen molecules, which split upon the metal surface into atoms. The hydrogen atoms then diffuse into the metal lattice to form PdH_x_ until equilibrium is reached, where x represents the atomic ratio of H to Pd. In this process, the lattice of Pd film expands and deflection occurs, thus reducing the cavity length *L*. The decrease in *L* leads to blueshift of the peaks or troughs of the FP interference fringe. Once the relationship between the H_2_ concentrations and the wavelength shift is established, the H_2_ concentration in the environment can be detected by monitoring the wavelength shift of the interference spectrum.

Figure 1b illustrates the schematic used to calculate the relationship between the deflection of Pd film and the H_2_ concentration. The Pd film can be considered thin and elastic. Initially the Pd film is assumed to be flat, and its position is indicated by the line AB in Figure 1b. After hydrogen absorption, the Pd film bends inwards as indicated by the arc AO’B, whose geometrical center is labeled as O. A simple formula Δ*λ*/*λ* = Δ*L*/*L* can be used to relate the wavelength shift Δ*λ* to the decrement of the cavity length Δ*L*. It can be seen from Figure 1b that Δ*L* equals the deflection value *h* of the Pd film. The deflection *h* is then related to the film strain *ε*_Pd_ caused by Pd lattice expansion [26], which can be expressed as follows:

(1) $$\varepsilon_{Pd}=\frac{R\beta -r}{r}=\frac{\beta -sin\beta }{sin\beta }$$

where *R* is the curvature radius of the Pd film after deflection, *r* is the inner radius of the capillary, and *β* is half of the AO’B arc angle.

The out-of-plane deflection *h* of the Pd film is related to the angle *β*:

(2) $$h=\frac{r\cdot sin\beta }{1+cos\beta }$$

Through Equations (1) and (2), using Taylor expansion, it can be obtained that

(3) $$h\approx \frac{\sqrt{6}}{2}r\sqrt{\varepsilon_{Pd}}$$

It is known that *ε*_Pd_ is related to H_2_ content via *ε*_Pd_ = 0.026*C*_H_ [4], where C_H_ is the concentration of hydrogen. Therefore, the relationship between the deflection *h* (i.e., cavity length change Δ*L*) and H_2_ concentration *C*_H_ can be obtained as follows:

(4) $$\Delta L=h=\frac{\sqrt{6}}{2}r\sqrt{0.026\cdot C_{H}}$$

Considering Δ*λ*/*λ* = Δ*L*/*L*, the wavelength shift Δ*λ* can be obtained as follows:

(5) $$\Delta \lambda =\lambda \cdot \frac{\sqrt{6}}{2L}r\sqrt{0.026\cdot C_{H}}$$

Thus, the relationship between the hydrogen concentration and the wavelength shift is established.

**Figure 2:**
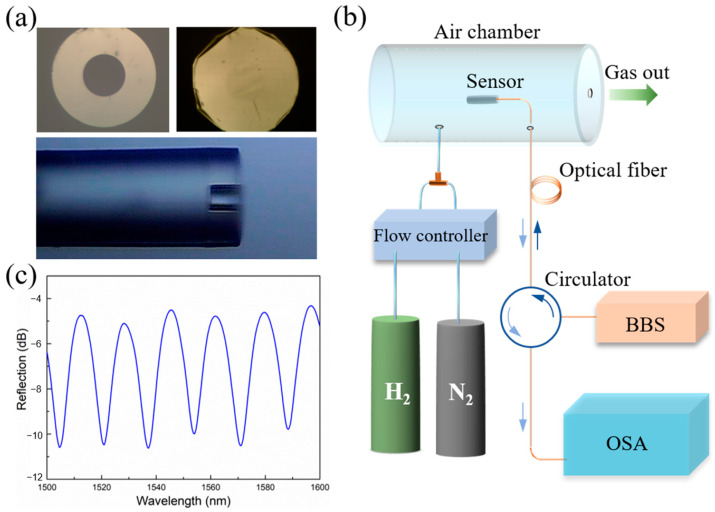
(**a**) Optical microscopy images of the fabricated sensor. Top: the capillary opening before (left) and after (right) the covering of the Pd thin film; bottom: side-view of the fabricated sensor. (**b**) The optical setup used to measure the performance of the sensors. H_2_ and N_2_ were mixed before being introduced into a home-made chamber. The concentration of the H_2_ was regulated by controlling its flow rate while keeping the total flow rate of H_2_ and N_2_ fixed at 300 sccm. BBS: broad-band source; OSA: optical spectrum analyzer. (**c**) shows the interference spectrum of the sensor.

The fabrication of the sensors consists of two steps: fusion of a section of silica capillary and transfer of a Pd thin film onto the open cavity (see more details in Section 3). Figure 2a shows the optical images of one fabricated sensor. It is clear that the Pd thin film was successfully transferred onto the silica capillary and completely covered the opening of the capillary. The outer diameter of the capillary is 125 μm, the same as that of single-mode fibers, making it easier for them to bond together. The capillary’s inner diameter is 50 μm, allowing sufficient surface area for welding. It was expected that the thickness of the Pd thin film would have a great influence on the performance of the sensor. Smaller thickness could lead to bigger bending of the film, but thinner films also make it harder to transfer them onto the capillary openings. After several preliminary experiments, we chose 30 nm thick Pd films to form the FP cavity H_2_ sensors. Figure 2c shows the interference spectrum of the sensor. Clear fringes were observed, suggesting good quality of the cavity. The length *L* of the Fabry–Pérot cavity can be calculated from the spectrum using the adjacent valley wavelengths [26]. It was estimated that the cavity was about 71 μm, which was close to what is estimated from Figure 2a. It is noted that the fringe contrast of the spectrum can be further improved by matching the reflection coefficient of the two surfaces. The reflection coefficient of the glass–air interface was smaller than that of the air–Pd interface. Therefore, it is feasible to deposit a metal layer on the fiber end to increase the reflection at this interface and thus enhance the fringe contrast as well as reduce the spectral width, which is helpful in boosting the performance of the sensor.

To test the H_2_ sensing performance of the suspended Pd film, the sensor was characterized using the setup shown in Figure 2b. There were two ports on the chamber for the inflow and outflow of the mixed gas of H_2_ and N_2_. A mass flow controller was used to regulate the H_2_ concentrations in the range 0 to 0.5%, with the total flow rate of the gas mixture fixed at 300 sccm.

**Figure 3:**
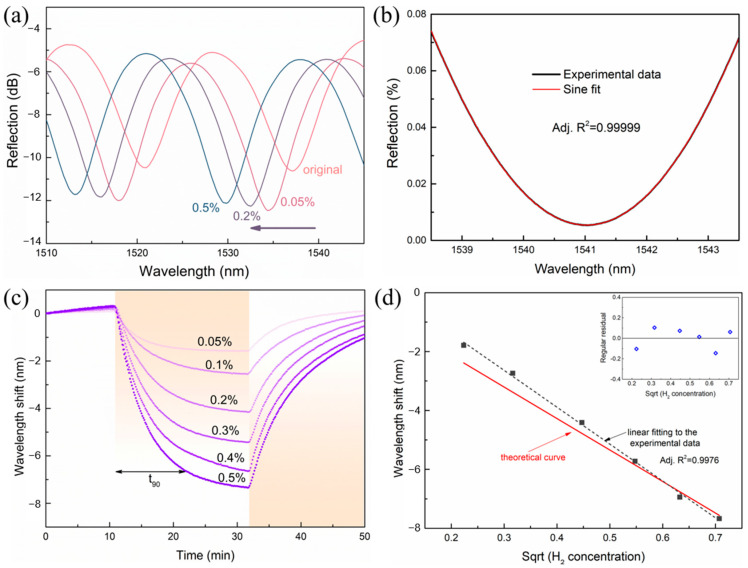
H_2_ sensing performance of the FP sensors. (**a**) Reflectance spectra of the sensor at 0.05%, 0.2%, and 0.5% H_2_ concentration. (**b**) Sine fitting of a reflectance spectrum to obtain the spectral position of the dip. (**c**) Time response and wavelength shift at different H_2_ concentrations. *t*_90_ is the response time of the sensor at 0.5% hydrogen concentration. (**d**) The relationship between the wavelength shift and square root of H_2_ concentration obtained from the experiments and the theoretical calculation. The *x*-axis used the square root of H_2_ concentration in order to obtain a linear fitting, as indicated from Equation (5) in the main text. The inset shows the regular residual of the linear fitting in panel (**d**).

Figure 3a shows the reflection spectra of the sensor that were recorded in equilibrium at 0.05%, 0.2%, and 0.5% H_2_ concentrations. As H_2_ was introduced to the gas chamber, the whole interference fringes of the sensor showed a consistent blue-shift, which became larger as the concentration of H_2_ increased. This can be ascribed to the strain-induced inward bending of the freestanding Pd film. To characterize this shift of the spectra caused by the adsorption of H_2_, we monitored the movement of one (at 1537.13 nm) of the dips, whose spectral positions were extracted using sine fitting to the dips as shown in Figure 3b. Considering the mediocre quality of the FP interference spectra, we used a sine function to find the spectral centroid instead of the Lorentz function used for high-quality FP cavities. The fitting result showed good agreement with the experimental results with R^2^ > 0.99.

Figure 3c shows the time response and wavelength shift of the sensor at various H_2_ concentrations between 0.05% and 0.5%. The sensor was initially kept in an N_2_ environment. As soon as H_2_ was introduced, the spectral positions of the dips progressively shifted to shorter wavelengths and eventually become stabilized. When the H_2_ concentration was as low as 0.05% (500 ppm), the wavelength shift was about 1.79 nm. As the H_2_ concentration increased to 0.5%, the wavelength shift was able to reach 7.67 nm. After the H_2_ inflow was stopped, the dip gradually returned to its original position, indicating the good recoverability of the suspended Pd membrane. It is estimated from Figure 3c that the response time of the sensor (defined as the time interval when the sensor reached 90% of its stable response) was about 11 min when the H_2_ concentration was 0.5%. The value of the response time varied a little bit under different H_2_ concentrations. Specifically, t90 decreased with the increases of H_2_ concentration, which was probably due to the variation of the diffusion speed of the hydrogen molecules. It is noted that the recovery time of the sensor was approximately the same as the response time. Although the response time and recovery time were in the range of minutes, the suspended Pd films did provide enhanced sensitivity, as shown below.

Figure 3d shows the relationship of the wavelength shift of the dip with the H_2_ concentration, together with the results obtained from the model in this paper. It is noted that the *x*-axis was the square root of the H_2_ concentration, which was intentionally used for a simple linear fitting, as indicated in Equation (5). As shown in Figure 3d, a good linear fitting was obtained for the experimental data, showing the good and robust response of the sensor to environmental hydrogen concentration changes. The red line in Figure 3d shows the calculation result from the theoretical model. Compared with the experimental data, the simple model provided a good prediction of performance of the sensor, indicating the rationality and accuracy of the modelling. It was expected that more accurate prediction can be obtained when advanced calculation methods are used to model mechanical strain in the Pd films as well as the deformation caused by the strain.

We then calculated the sensitivity of the proposed sensor by taking the ratio between the wavelength shift and the corresponding H_2_ concentration [29]. At H_2_ concentration of 500 ppm, the corresponding sensitivity of H_2_ detection was about 3.6 pm/ppm, and the sensitivity slightly decreased as the hydrogen concentration increased. The sensitivity at 0.5% H_2_ concentration was 1.5 pm/ppm. We monitored the wavelength fluctuation (noise level) of the sensor at stable H_2_ concentrations and calculated the standard deviation of the fluctuation (1*σ* = 11.6 pm). Then, the limit of detection (LOD) of the H_2_ sensor was LOD $=\frac{\sigma }{\mathrm{sensitivity}}=\frac{11.6\mathrm{pm}}{3.6\mathrm{pm}/\mathrm{ppm}}\approx$ 3.3 ppm.

Sensing performance of other reported H_2_ sensors based on FP cavities is shown in Table 1. It was found that the proposed sensors with freestanding Pd membranes presented in this work were able to achieve relatively high sensitivity (3.6 pm/ppm) at a low hydrogen concentration (500 ppm), which can mainly be attributed to the fact that suspended Pd thin films were used as both the active sensing materials and the reflective surfaces of the FP cavities. In other reported sensors, the supporting composite membranes were usually made of materials with large stiffness, which weakens the deflection caused by the internal strain of the Pd film. For example, the Young’s modulus of graphene is nearly ten times of that of Pd, even though graphene can be made into one-atom thickness. Without any supporting layers, the deflection of the cavities is only determined by the material (Young’s modulus) and geometrical (moment of inertia) properties of the suspended Pd film. While the response time and recovery time were larger than some of the values in Table 1, the proposed sensor did provide excellent sensitivity and a detection limit, making it desirable for applications where high sensitivity is preferred. It is known that the alloying of Pd offers a plausible means to improve the response of the H_2_ sensor [30]. In particular, it is shown that mixing Pd with Au facilitates the hydrogen absorption process in the metal [31]. Therefore, instead of pure Pd thin films, suspended Pd/Au alloy thin films could be used in the proposed sensor to improve its response time. Other metals, such as silver and copper, can also be used for this purpose. Such topics will be explored in a future study. We would like to emphasize that the miniaturized sensor with a small footprint can be easily fit into sensing application in a confined space. The integration with optical fibers also enables remote sensing, separating the active sensing area and the data analysis units.

**Figure 4:**
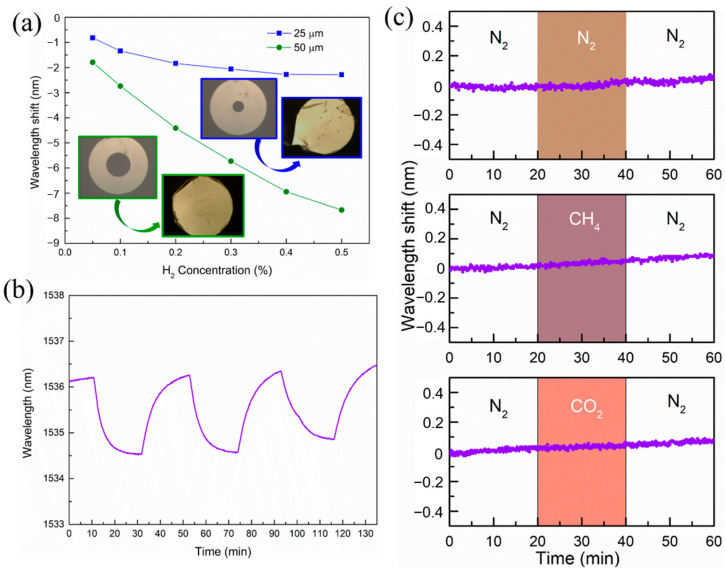
Other factors considered in the design of the H_2_ sensors. (**a**) Comparison of the sensing performance of H_2_ sensors made from 25 and 50 μm apertures. The insets show the SEM images of the capillary ends before and after the covering of the Pd thin films. (**b**) Stability test of the sensor. (**c**) The specificity test of the sensor showing no response to N_2_, CH_4_, and CO_2_ gases with 0.5% concentration.

We also studied the effect of capillary aperture size on the sensitivity of the sensor. In Figure 4a, we compare the wavelength shifts of sensors made from 25 μm and 50 μm capillary apertures at concentrations below 0.5% H_2_. With smaller aperture, the wavelength shift became smaller. At H_2_ concentration of 0.4%, the wavelength shift from the FP sensor with 50 μm aperture was ≈3.4 times larger than that with 25 μm aperture. Additionally, larger aperture leads to a larger linear measurement range. Based on the model in Equation (4), there is a positive correlation between the Pd film deflection value h and the capillary’s aperture (2*r*). The larger the capillary aperture, the greater the Pd film deflection (FP cavity length change), and therefore the higher the sensor’s sensitivity.

Figure 4b shows the variation of the spectral positions of the dip when the sensor was exposed to N_2_ and H_2_ (500 ppm) consecutively for several cycles. It can be seen that the sensor showed good repeated stability. The specificity of the sensor to H_2_ gas was also confirmed by carrying out the same experiments with 0.5% concentration of N_2_, CH_4_, and CO_2_ gases, and the test results are shown in Figure 4c. It is clear that the sensor showed no responses to the CO_2_, CH_4_, and N_2_ gases, which meets the performance standard of hydrogen sensors [33].

## 3. Materials and Methods

### 3.1. Fabrication of the Sensor

A silica capillary (Polymicro, TSP025150) with a 50 μm aperture was fused to a conventional single-mode fiber using a fusion splicer (Fujikura, FSM-45PM, Tokyo, Japan, slice parameters: 50 bits, 150 ms). The capillary was then cut at a distance of *L* from the splice joint using a standard fiber cutter under an optical microscope. This distance *L* determined the initial length of the FP cavity, whose value can be adjusted from 20 to 100 μm.

**Figure 5:**
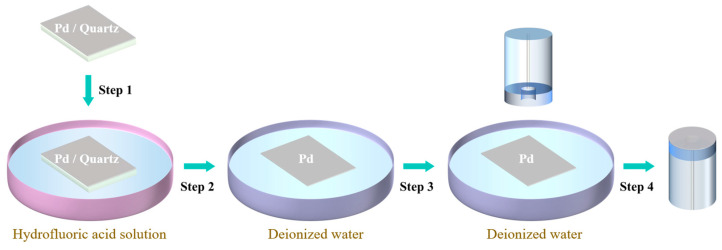
Schematic diagram of the transfer process of Pd thin films onto a silica capillary. The capillary was fused onto the end of a single-mode fiber.

A Pd film with a thickness of 30 nm was prepared on quartz substrate by electron beam evaporation. Then, the Pd film was transferred onto the capillary to form a microcavity. The transfer process is schematically shown in Figure 5. First, the Pd/quartz sample was immersed in hydrofluoric acid solution to separate the Pd film from (step 1 in Figure 5). Then, the Pd film was moved to deionized water using a clean slide, and the process was repeated three times to wash off residual ions (step 2 in Figure 5). Finally, the Pd film floating on the water surface was transferred onto the open end of the microcavity by a dipping process (step 3 in Figure 5). The optical fiber was mounted on a translation stage with the capillary facing down the water and moved slowly towards the Pd film. Once the capillary touched the Pd film, the optical fiber was pulled up. Due to the surface tension of water, the Pd film was attached to the capillary, forming a micro cavity (step 4 in Figure 5). So far, the preparation of the Pd nanofilm optical fiber sensor has been completed.

### 3.2. H_2_ Sensing Tests

The sensor was characterized using the setup shown in Figure 2b. A broadband optical source (BBS, operating wavelength: 1250 nm~1650 nm) was used in the experiments, and an optical spectrum analyzer (OSA, Golight, AE8600) was used to record and monitor the shift of the reflection spectra of the sensors. The detection wavelength resolution was 0.02 nm.To test the sensor’s response, initially only N_2_ was introduced into the gas chamber with one mass flowrate controller. Then, H_2_ was introduced at a fixed concentration until the reflection spectrum became stable. The H_2_ concentration was regulated by controlling the relative ratio of the flowrates of the two gases while keeping the total fixed at 300 sccm. After certain time, H_2_ was turned off and N_2_ was injected into the gas chamber again to return to the initial state. During the tests, the reflection spectra of the sensor were recorded across a broad wavelength range or the spectra near one of the dips (≈1537.13 nm in this case) were collected every 8 s to monitor the spectral shift.

## 4. Conclusions

In this study, we proposed and demonstrated a highly sensitive optical fiber H_2_ sensor based on a FP cavity consisting of a suspended Pd membrane. The Pd film acted both as a reflective surface of the FP cavity and the active H_2_ sensing material. Upon H_2_ absorption, the strain inside the Pd film caused deflection of the film, leading to wavelength shift of the spectra. The magnitude of the shift depended on the deflection and thus the H_2_ concentration. High H_2_ sensitivity at low concentrations was achieved. At a low H_2_ concentration of 500 ppm, the wavelength shift of the sensor was able to reach 1.79 nm, corresponding to a sensitivity of about 3.6 pm/ppm and a detection limit about 3.3 ppm. The sensor showed good cycle stability and gas selectivity. The FP cavity H_2_ sensors with suspended Pd thin films and no other supporting materials provided a compact all-optical solution for high sensitivity detection in low-hydrogen environments.

## Figures and Tables

**Table 1:** Performance of several Fabry–Pérot interferometer hydrogen sensors [19,20,21,26,32].

Sensitive Film	Detection Range	Sensitivity and Detection Limit	Response Time	Recovery Time	Working Temp.	Ref.
100 μm Pd	4–10%	0. 144 pm/ppm at 8% C_H_ (500 ppm detection limit)	401 s	≈500 s	RT	[32]
2 μm Pd	0.5–5%	(32 ppm detection limit)	≈30 min	-	RT	[19]
50 nm Pd and 2 nm Ni	4%	0.0175 pm/ppm at 4% C_H_	50 s	-	RT	[20]
Multiple 20 nm Pd	2–8%	≈0.0019 pm/ppm at 8% C_H_	≈2 min	≈5 min	RT	[21]
5.6 nm Pd and 3 nm MLG	0.02–3%	0.25 pm/ppm at 0.02% C_H_ (20 ppm detection limit)	18 s	-	RT	[26]
30 nm Pd	0.05–0.5%	3.6 pm/ppm at 0.05% C_H_ 1.5 pm/ppm at 0.5% C_H_ (3.3 ppm detection limit)	11 min	11 min	RT	This work

## Data Availability

Data are available from the authors upon request.

## Sample Availability

Samples of the compounds are not available from the authors.
